# Identifying the best candidates for reduced port gastrectomy

**DOI:** 10.1007/s10120-023-01438-6

**Published:** 2023-10-24

**Authors:** Jae Hun Chung, Jawon Hwang, Sung Hyun Park, Ki-Yoon Kim, Minah Cho, Yoo Min Kim, Hye Jung Shin, Si-Hak Lee, Sun-Hwi Hwang, Woo Jin Hyung, Hyoung-Il Kim

**Affiliations:** 1 Division of Gastrointestinal Surgery, Department of Surgery, Pusan National University Yangsan Hospital, Yangsan, Republic of Korea; 2 Research Institute for Convergence of Biomedical Science and Technology, Pusan National University Yangsan Hospital, Yangsan, Republic of Korea; 3 School of Medicine, Pusan National University, Busan, Republic of Korea; 4 Department of Surgery, Yonsei University College of Medicine, 50-1 Yonsei-Ro Seodaemun-Gu, Seoul, 03722 Republic of Korea; 5 Gastric Cancer Center, Yonsei Cancer Center, Yonsei University Health System, Seoul, Republic of Korea; 6 Biostatistics Collaboration Unit, Department of Biomedical Systems Informatics, Yonsei University College of Medicine, Seoul, Republic of Korea

**Keywords:** Gastrectomy, Gastric cancer, Minimally invasive surgery, C-reactive protein

## Abstract

**Background:**

Previous studies have focused on the non-inferiority of RPG compared with conventional port gastrectomy (CPG); however, we assumed that some candidates might derive more significant benefit from RPG over CPG.

**Methods:**

We retrospectively analyzed the clinicopathological and perioperative parameters of 1442 patients with gastric cancer treated by gastrectomy between 2009 and 2022. The C-reactive protein level on postoperative day 3 (CRPD3) was used as a surrogate parameter for surgical trauma. Patients were grouped according to the extent of gastrectomy [subtotal gastrectomy (STG) or total gastrectomy (TG)] and lymph node dissection (D1+ or D2). The degree of surgical trauma, bowel recovery, and hospital stay between RPG and CPG was compared among those patient groups.

**Results:**

Of 1442 patients, 889, 354, 129, and 70 were grouped as STGD1+, STGD2, TGD1+, and TGD2, respectively. Compared with CPG, RPG significantly decreased CRPD3 only among patients in the STGD1+ group (CPG: *n* = 653, 84.49 mg/L, 95% CI 80.53–88.45 vs. RPG: n = 236, 70.01 mg/L, 95% CI 63.92–76.09, *P* < 0.001). In addition, the RPG method significantly shortens bowel recovery and hospital stay in the STGD1+ (*P* < 0.001 and *P* < 0.001), STGD2 (*P* < 0.001 and *P* < 0.001), and TGD1+ (*P* = 0.026 and *P* = 0.007), respectively. No difference was observed in the TGD2 group (*P* = 0.313 and *P* = 0.740).

**Conclusions:**

The best candidates for RPG are patients who undergo STGD1+, followed by STGD2 and TG D1+, considering the reduction in CRPD3, bowel recovery, and hospital stay.

**Supplementary Information:**

The online version contains supplementary material available at 10.1007/s10120-023-01438-6.

## Introduction

Minimally invasive surgery (MIS) has radically transformed surgical outcomes by reducing surgical trauma, improving patient recovery, and shortening the lengths of hospital stays [1–7]. To minimize invasiveness, reduced port laparoscopic surgery (RPS) techniques are applied more frequently [8]. Recent technical developments were initially developed for the surgical treatment of benign diseases; however, they have allowed RPS techniques to be applied to treat malignant diseases, such as colorectal and gastric cancers [9–12]. The use of reduced port gastrectomy (RPG) is increasing [11, 13–15], and prior studies have reported the non-inferiority of RPG compared with conventional port gastrectomy (CPG) regarding lymph node dissection (LND), bleeding, and pain after surgery [14]. However, the widespread adoption of RPG remains limited by the lack of specialized instruments, a constrained operating view, restricted instrument movement, and longer operating times [14]. Furthermore, RPG can be applied to various indications; however, the patient subgroups that would benefit most from RPG remain unclear. The goals of RPG are minimized surgical stress and expedited recovery. Therefore, quantitative parameters must be identified to measure the effects of RPG and assess surgical trauma to determine which patients would benefit most from RPG over CPG.

Notably, several established clinical laboratory markers evaluate systemic inflammation in daily clinical practice, including C-reactive protein (CRP) and the cytokines interleukin 1, interleukin 6, and tumor necrosis factor-alpha [16, 17]. CRP is the most specific and sensitive marker for assessing the extent of surgery-induced tissue injury and is well suited for evaluating whether RPG can reduce surgical stress [18]. The ability to predict surgical stress using a quantitative parameter, such as CRP, would enable surgeons to select either CPG or RPG depending on which gastrectomy approach would be most beneficial for each patient.

## Methods

### Patients

**Fig. 1:**
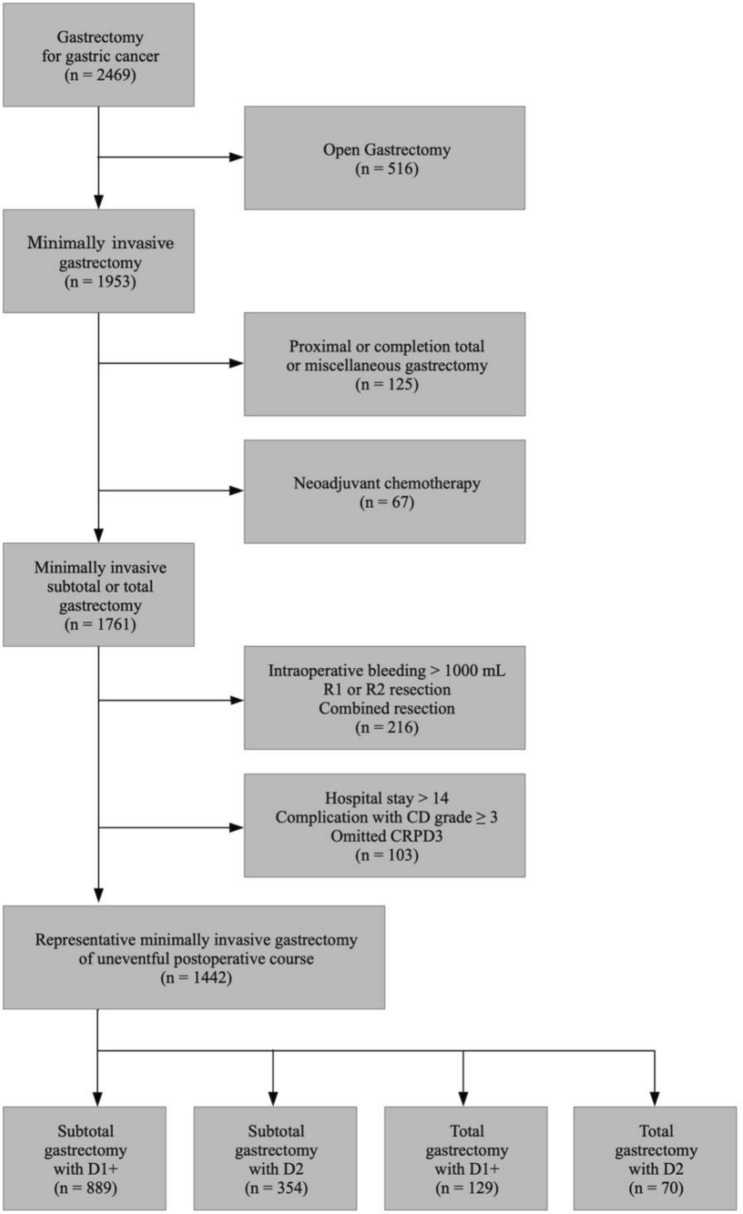
Flowchart of inclusion and exclusion criteria

A total of 2469 patients with gastric cancer were treated with gastrectomy by a single surgeon (Hyoung-Il Kim) at Yonsei Cancer Center between 2009 and 2022. We collected retrospective data on clinicopathological and perioperative parameters to examine their impacts on surgical trauma. In this study, we assumed that the level of CRP on postoperative day 3 (CRPD3) is a surrogate parameter for the total surgical stress associated with the procedures [19–22]. To focus our analysis on ideal MIS conditions, we excluded patients with a history of (1) open gastrectomy or an MIS gastrectomy that was converted to an open gastrectomy (*n* = 516); (2) proximal gastrectomy, completion total gastrectomy (TG), or miscellaneous gastrectomy (*n* = 125); (3) neoadjuvant chemotherapy (*n* = 67); or (4) intraoperative bleeding over 1000 cc, over 15 days of hospital stay, Clavien–Dindo classification grade 3 or higher, or missing CRPD3 values (*n* = 319). The remaining 1442 patients were enrolled in the final statistical analysis (Fig. 1). Informed consent was waived because of the study’s retrospective nature, and the analysis used anonymous clinical data. This study was approved by the Institutional Review Board of Yonsei University College of Medicine, Korea (IRB No. 4-2022-1543).

### Port insertion protocol for CPG and RPG

CPG was performed through a 10 mm port located in the umbilical area for the laparoscope, a 12 mm port located in the right lower quadrant area, and the left paramedian area. A 5 mm port is located in both the right upper quadrant area and the left upper quadrant area. RPG was performed using an umbilical incision with a 12 mm port at the RUQ as an assist port. The umbilical incision size for both the CPG and RPG approaches remained consistently 25–30 mm. In CPG, a 10 mm port was initially employed for laparoscopy and extended to 25–30 mm at the conclusion of the procedure for specimen extraction. Conversely, for RPG, a 25–30 mm incision was initially created from the beginning to accommodate the multiport configuration.

Special instruments, SurgiTractor (St0306kb, SurgiCore Co., Ltd, Korea), ArtiSential fenestrated forceps, and a medium–large clip applier (AUF01-L and ACA01-L, LivsMed, Seongnam, Korea) were used for RPG in laparoscopic gastrectomy [23]. For robotic RPG, Single-Site system and associated instruments [Cadiere forceps and wristed needle drive (478055 and 478115, Intuitive Surgical, Sunnyvale, CA, USA)] were used through Single-Site port (478065, intuitive Surgical, Sunnyvale, CA, USA) as in a previously published study [24].

### CRPD3 analyses by subgroup and associations with perioperative parameters

**Fig. 2:**
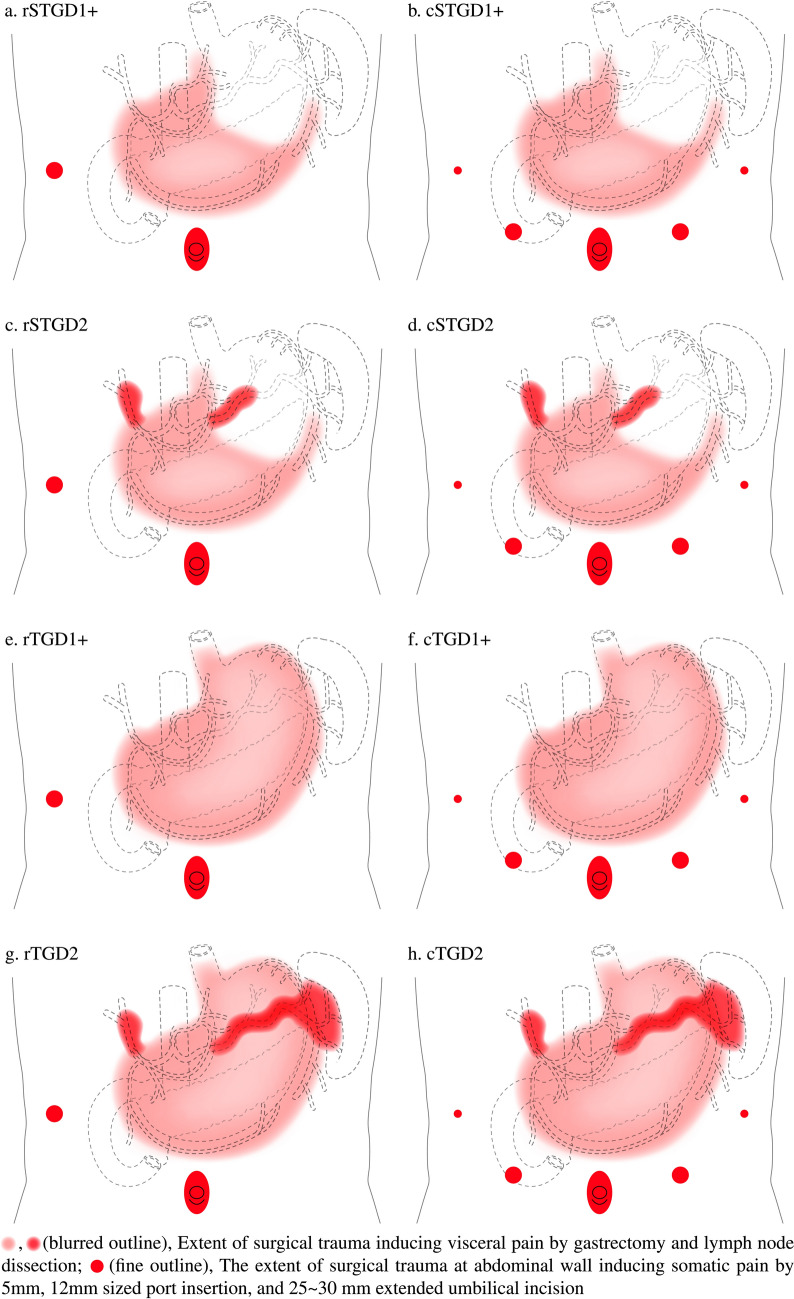
Schematic illustration of surgical trauma following reduced port gastrectomy or conventional port gastrectomy according to the extent of lymph node dissection and gastrectomy

The patients were categorized according to gastrectomy type [subtotal gastrectomy (STG) or TG] and the extent of LND (D1+ or D2), resulting in four patient groups: STGD1+ (*n* = 889), STGD2 (*n* = 354), TGD1+ (*n* = 129), and TGD2 (*n* = 70). Patients were further subdivided based on whether they received RPG (*n* = 305, 21.15%) or CPG (*n* = 1137, 78.85%). Figure 2 illustrates surgical trauma following RPG or CPG according to the extent of LND and gastrectomy. We analyzed CRPD3 values for all groups and estimated the contributions of several perioperative parameters to CRPD3 values using linear regression analysis.

### Statistics

Descriptive statistics are presented as the mean and standard deviation for continuous variables and the number and percent for categorical variables. Student’s *t* test and the Chi-square test or Fisher exact test were performed to evaluate differences in continuous and categorical variables. Linear regression was applied to assess associations between postoperative serum CRP levels and potential risk factors. Variables with *P* < 0.05 in the univariate analysis were examined in a multivariate linear regression analysis to estimate the contributions of perioperative parameters to CRPD3. All statistical analyses were conducted using SAS software version 9.4 (SAS Institute, Cary, NC) and R package (v. 4.0.4, http://www.r-project.org/). The graphs were produced using GraphPad Prism software version 10.0.2 for Mac (GraphPad Software, San Diego, CA, USA). Reported *P*-values are two-sided, and statistical significance was set at *P* < 0.05.

## Results

### Patients

The clinical characteristics of patients in the CPG and RPG subgroups are compared in Table 1. No significant differences were observed between these two groups for clinical features, including sex (*P* = 0.059), body mass index (BMI; CPG: 23.88 ± 3.32 kg/m^2^ vs. RPG: 23.66 ± 3.05 kg/m^2^, *P* = 0.315), American Society of Anesthesiologists Physical Status (ASA-PS; *P* = 0.346), operating time (minutes) (CPG: 166.31 ± 57.70 vs. RPG: 161.80 ± 39.93, *P* = 0.115), diabetes mellitus (DM, *P* = 0.302), non-tuberculosis (TB) pulmonary disease (*P* > 0.999), nephrology disease (*P* = 0.491), liver disease (*P* = 0.816), cerebral disease (*P* = 0.457), or TB (*P* = 0.770). However, significant differences between groups were observed for hypertension (HTN, *P* = 0.020) and cardiology disease (*P* = 0.046). According to pathologic reports, patients who underwent CPG had more advanced forms of gastric cancer than those who underwent RPG, as assessed by T-stage (*P* = 0.003), N-stage (*P* = 0.042), and total stage (*P* = 0.002). As a result, the CPG group had more extensive LND (*P* < 0.001), gastrectomy (*P* < 0.001), and omentectomy (*P* = 0.002) than the RPG group.

**Table 1:** Clinical characteristics of patients in the conventional port gastrectomy group compared with those in the reduced port gastrectomy group

Clinical characteristics	CPG (*n* = 1137)		RPG (*n* = 305)		*P* value
	Mean or *N*	SD or %	Mean or *N*	SD or %	
Age (years)	60.43	11.72	57.56	12.48	0.0002
Sex					0.0588
Male	716	62.97	174	57.05	
Female	421	37.03	131	42.95	
BMI (kg/m^2^)	23.88	3.32	23.66	3.05	0.3150
Operating time (min)	166.31	57.70	161.80	39.93	0.1152
ASA-PS					0.0370
< 2	277	24.36	57	18.69	
≥ 2	860	75.64	248	81.31	
Underlying disease					
HTN	413	36.32	89	29.18	0.0201
DM	192	16.89	44	14.43	0.3024
Non-TB Pulmonary disease	18	1.58	4	1.31	> 0.9999
Cardiology disease	71	6.24	10	3.28	0.0458
Nephrology disease	9	0.79	4	1.31	0.4912
Liver disease	52	4.57	13	4.26	0.8161
Cerebral disease	31	2.73	6	1.97	0.4565
TB	73	6.42	21	6.89	0.7703
Depth of invasion					0.0028
T1	863	75.90	256	83.93	
T2, T3 or T4	274	24.10	49	16.07	
Node status					0.0415
N0	915	80.47	261	85.57	
N1, N2, or N3	222	19.53	44	14.43	
Stage					0.0020
I	907	79.77	267	87.54	
II, III	230	20.23	38	12.46	
Extent of LND					< 0.0001
D1+	771	67.81	247	80.98	
D2	366	32.19	58	19.02	
Extent of Gastrectomy					< 0.0001
Distal gastrectomy	958	84.26	285	93.44	
Total gastrectomy	179	15.74	20	6.56	
Extent of Omentectomy					0.0018
Partial omentectomy	994	87.42	286	93.77	
Total omentectomy	143	12.58	19	6.23	

*ASA-PS* American Society of Anesthesiologists Physical Status, *BMI* body mass index, *CPG* conventional port gastrectomy, *DM* diabetes mellitus, *HTN* hypertension, *LND* lymph node dissection, *N* node, *RPG* reduced port gastrectomy, *SD* standard deviation, *T* tumor, *TB* tuberculosis

### CRPD3, bowel recovery, and hospital stay in each subgroup

**Fig. 3:**
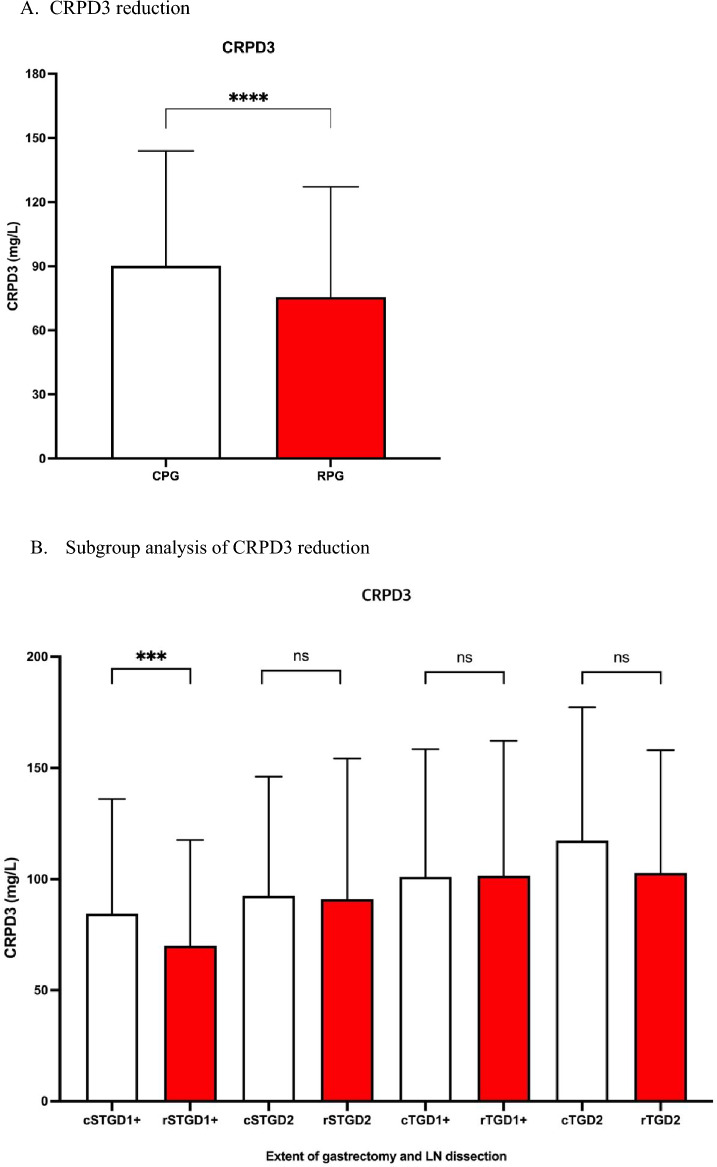

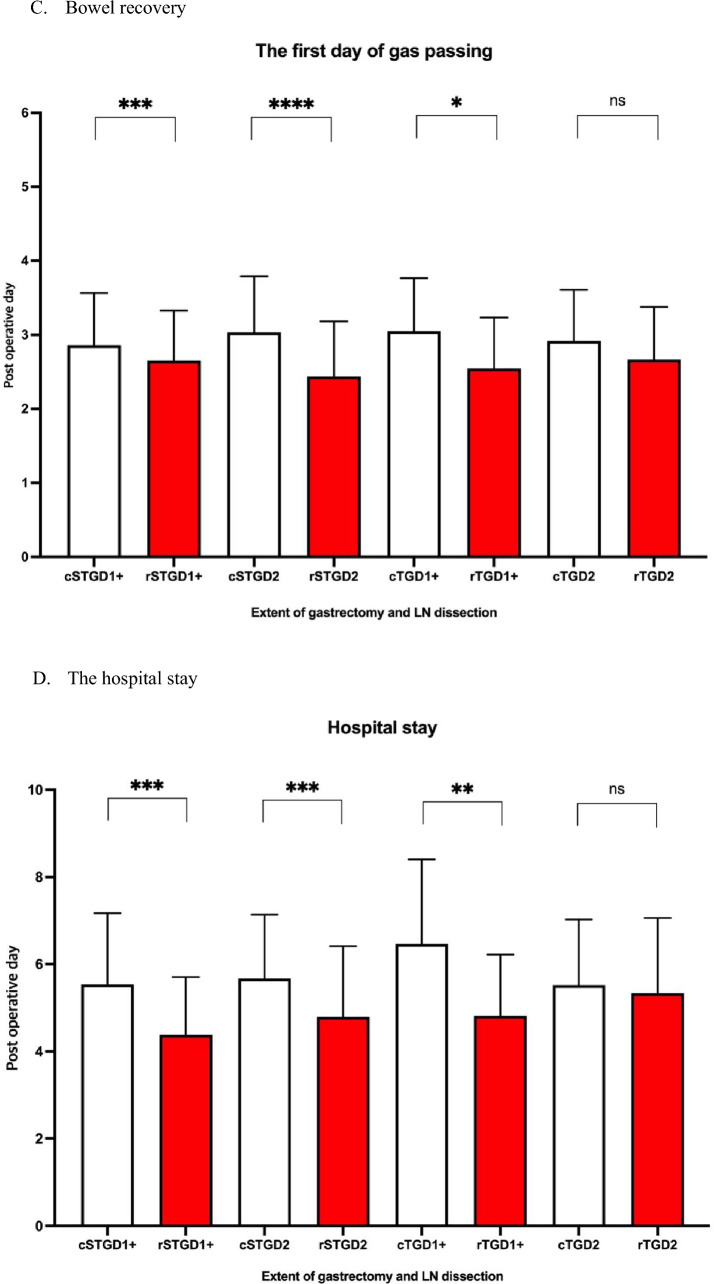
Subgroup analysis identifying the advantage of reduced port gastrectomy. **a** CRPD3 reduction. **b** Subgroup analysis of CRPD reduction. **c** Bowel recovery. **d** The hospital stay. *CPG* conventional port gastrectomy, *RPG* reduced port gastrectomy, *cSTGD1*+ conventional port subtotal gastrectomy with D1+ dissection, *rSTGD1*+ reduced port subtotal gastrectomy with D1+ dissection, *cSTGD2* conventional port subtotal gastrectomy with D2 dissection, *rSTGD2* reduced port subtotal gastrectomy with D2 dissection, *cTGD1*+ conventional port total gastrectomy with D1+ dissection, *rTGD1*+ reduced port total gastrectomy with D1+ dissection, *cTGD2* conventional port total gastrectomy with D2 dissection, *rTGD2* reduced port total gastrectomy with D2 dissection

The RPG method significantly decreased CRPD3 values compared to the CPG method. (CPG: *n* = 1137, 90.13 mg/L, 95% CI 86.87–93.30 vs. RPG: *n* = 305, 75.49 mg/L, 95% CI 63.95–81.69, *P* < 0.001) (Fig. 3a). In subgroup analysis, the RPG method significantly decreased CRPD3 values in the STGD1+ group (CPG: *n* = 635, 84.49 mg/L, 95% CI 80.53–88.45 vs. RPG: *n* = 236, 70.01 mg/L, 95% CI 63.92–76.09, *P* < 0.001*,* Fig. 3b). No significant advantages of using RPG over CPG were observed for the STGD2 (CPG: *n* = 305, 92.51 mg/L, 95% CI 86.48–98.55 vs. RPG: *n* = 49, 91.03 mg/L, 95% CI 73.06–109.01, *P* = 0.861), TGD1+ (CPG: *n* = 118, 101.11 mg/L, 95% CI 90.66–111.56 vs. RPG: *n* = 11, 101.50 mg/L, 95% CI 60.72–142.28, *P* = 0.983), or TGD2 (CPG: *n* = 61, 117.35 mg/L, 95% CI 102.02–132.70 vs. RPG: *n* = 9, 102.79 mg/L, 95% CI 60.33–145.25 *P* = 0.495) groups.

The RPG method significantly shortens bowel recovery in the STGD1+ (CPG: *n* = 651, 2.86 days, 95% CI 2.81–2.92vs. RPG: n = 236, 2.66 days, 95%CI, 2.57–2.74, *P* < 0.001, Fig. 3c), STGD2(CPG: *n* = 305, 3.03 days, 95% CI 2.95–3.12 vs. RPG: *n* = 48, 2.44 days, 95% CI 2.22–2.65, *P* < 0.001), and TGD1+ (CPG: *n* = 118, 3.05 days, 95% CI 2.92–3.18 vs. RPG: *n* = 11, 2.55 days, 95% CI 2.08–3.01, *P* = 0.026) groups. There was no significant benefit of RPG compared with CPG for the TGD2 group (CPG: *n* = 61, 2.92 days, 95% CI 2.74–3.10 vs. RPG: *n* = 9, 2.67 days, 95% CI 2.12–3.21, *P* = 0.313).

Similar to the above results, the RPG method significantly shortens the hospital stay in the STGD1+ (CPG: *n* = 653, 5.53 days, 95% CI 5.41–5.66 vs. RPG: *n* = 236, 4.39 days, 95% CI 4.21–4.56, *P* < 0.001, Fig. 3d), STGD2 (CPG: *n* = 305, 5.67 days, 95% CI 5.50–5.84 vs. RPG: *n* = 49, 4.80 days, 95% CI 4.33–5.26, *P* < 0.001), and TGD1+ (CPG: *n* = 118, 6.47 days, 95% CI 6.11–6.82 vs. RPG: *n* = 11, 4.82 days, 95% CI 3.88–5.76, *P* = 0.007) groups. No significant advantages of using RPG over CPG were observed for the TGD2 group (CPG: *n* = 56, 5.52 days, 95% CI 5.11–5.92 vs. RPG: *n* = 9, 5.33 days, 95% CI 4.00–6.67, *P* = 0.740).

### Quantitative estimation of the impacts of perioperative parameters on CRPD3

On univariate analysis (Table 2), the following factors were significantly associated with increasing CRPD3: male sex, age ≥ 65 years, BMI ≥ 25 kg/m^2^, ASA-PS ≥ 2, CPG, D2 dissection, T-stage > 1, TG, total omentectomy, node metastasis, HTN, and DM. Multivariate analysis was performed to estimate each parameter’s contributions to changes in CRPD3. Significant factors associated with increasing CRPD3 were male sex (*P* < 0.001, reference value female sex), BMI ≥ 25 kg/m^2^ (*P* < 0.001, reference value BMI < 25 kg/m^2^), CPG (*P* = 0.0109, reference value RPG), D2 dissection (*P* = 0.0174, reference value D1+ dissection), TG (*P* < 0.001, reference value STG), total omentectomy (*P* = 0.042, reference value partial omentectomy), HTN (*P* < 0.001), and DM (*P* = 0.0138). Finally, linear regression was performed to estimate CRPD3 levels after minimally invasive gastrectomy to treat gastric cancer (Table 3). Every variable included in the linear regression model was significant: male sex (32.56 ± 2.79 mg/L, *P* < 0.001), BMI ≥ 25 kg/m^2^ (10.21 ± 2.94 mg/L, *P* < 0.001), RPG (− 8.97 ± 3.39 mg/L, *P* = 0.008), D2 dissection (9.26 ± 3.10 mg/L, *P* = 0.003), TG (14.79 ± 4.97 mg/L, *P* < 0.001), total omentectomy (9.53 ± 4.67 mg/L, *P* = 0.041), HTN (11.84 ± 2.98 mg/L, *P* < 0.001), and DM (9.66 ± 3.85 mg/L, *P* = 0.012). The pie chart shows the proportional contribution of each parameter on the CRPD3. (Electronic Supplementary Material, Fig. 1).

**Table 2:** Univariate and multivariate analysis of clinical factors affecting CRPD3 by linear regression

Clinical factors		Univariate analysis			Multivariate analysis		
		Coefficient	Se	*P* value	Coefficient	SE	*P* value
Sex	Female	Ref		–	Ref		–
	Male	37.25	2.74	< 0.0001	32.61	2.80	< 0.0001
Age (years)	< 65	Ref		–	Ref		–
	≥ 65	9.98	2.93	0.0007	2.65	2.95	0.3691
BMI (kg/m^2^)	< 25	Ref		–	Ref		–
	≥ 25	17.03	3.07	< 0.0001	10.18	2.94	0.0006
ASA-PS	< 2	Ref		–	Ref		–
	≥ 2	7.59	3.35	0.0234	− 1.12	3.33	0.7362
Procedure	CPG	Ref		–	Ref		–
	RPG	− 14.64	3.44	< 0.0001	− 8.69	3.41	0.0109
Extent of LND	D1+	Ref		–	Ref		–
	D2	12.89	3.08	< 0.0001	8.37	3.52	0.0174
Extent of gastrectomy	STG	Ref		–	Ref		–
	TG	22.22	4.06	< 0.0001	14.94	4.02	0.0002
Extent of omentectomy	p-Omn	Ref		–	Ref		–
	t-Omn	27.03	4.42	< 0.0001	9.51	4.67	0.0420
EGC vs. AGC	EGC	Ref		–	Ref		–
	AGC	7.30	3.39	0.0313	− 1.70	3.88	0.6617
Node metastasis	N0	Ref		–	Ref		–
	> N0	8.38	3.64	0.0215	4.85	4.00	0.2257
HTN	(−)	Ref		–	Ref		–
	(+)	18.42	2.93	< 0.0001	11.54	3.16	0.0003
DM	(−)	Ref		–	Ref		–
	(+)	20.64	3.78	< 0.0001	9.55	3.88	0.0138
Pulmonary disease	(−)	Ref		–			
	(+)	14.21	11.53	0.2178			
Cardiologic disease	(−)	Ref		–			
	(+)	5.24	6.14	0.3934			
Nephrological disease	(−)	Ref		–			
	(+)	− 11.92	14.95	0.4256			
Liver disease	(−)	Ref		–			
	(+)	− 6.52	6.81	0.3388			
Cerebral disease	(−)	Ref		–			
	(+)	− 2.84	8.94	0.7504			
TB	(−)	Ref		–			
	(+)	0.61	5.73	0.9145			

*AGC* advanced gastric cancer, *ASA-PS* American Society of Anesthesiologists Physical status, *BMI* body mass index, *CPG* conventional port gastrectomy, *CRPD3* C-reactive protein level on postoperative day 3, *DM* diabetes mellitus, *EGC* early gastric cancer, *HTN* hypertension, *LND* lymph node dissection, *p-Omn* partial omentectomy, *t-Omn* total omentectomy, *RPG* reduced port gastrectomy, *ref* reference variable, *SE* standard error, *STG* subtotal gastrectomy, *TB* tuberculosis, *TG* total gastrectomy

**Table 3:** Estimated contribution of parameters on CRPD3 by linear regression

Clinical factors		Result of linear regression		
		Coefficient	SE	*P* value
Sex	Female	Ref		–
	Male	32.56	2.78	< 0.001
BMI (kg/m^2^)	< 25	Ref		–
	≥ 25	10.21	2.94	< 0.001
Procedure	CPG	Ref		–
	RPG	− 8.97	3.39	0.008
Extent of LND	D1+	Ref		–
	D2	9.26	3.10	0.003
Extent of gastrectomy	STG	Ref		–
	TG	14.79	3.97	< 0.001
Extent of omentectomy	p-Omn	Ref		–
	t-Omn	9.53	4.67	0.041
HTN	(−)	Ref		–
	(+)	11.84	2.98	< 0.001
DM	(−)	Ref		–
	(+)	9.66	3.85	0.012
Constant		54.02	2.73	< 0.001

*BMI* body mass index, *CPG* conventional port gastrectomy, *DM* diabetes mellitus, *HTN* hypertension, *LND* lymph node dissection, *p-Omn* partial omentectomy, *ref* reference variable *RPG* reduced port gastrectomy, *SE* standard error, *STG* subtotal gastrectomy, *TG* total gastrectomy, *t-Omn* total omentectomy

## Discussion

Since the initial application of RPS techniques for gastrectomy [11], many advantages of the method have been reported [25–29]. However, a detailed understanding of the patient groups most suited to receive RPG is yet to be determined. This study identified CRPD3 as an easily measured clinical laboratory parameter that could estimate surgical trauma and predict postoperative recovery. Serum CRP is an acute-phase reactant that responds to inflammation and tissue damage. The postoperative serum CRP concentration is a quantitative index that integrates the cumulative effects of preoperative comorbidities, surgical invasion, surgical duration, anesthetic management, and analgesia [30, 31]. CRPD3 has been used in many previous studies as a quantitative indicator of surgical stress that predicts the postoperative recovery course [19–22], and CRPD3 values correlate with the length of hospital stay, short-term recovery, and postoperative complications [32]. This study’s results corroborate with previous reports. The levels of CRP were highest on postoperative day 3. Therefore, we selected CRPD3 as a surrogate parameter to reflect the degree of surgical damage (Electronic Supplementary Material, Fig. 2a). Pearson’s correlation coefficients analysis of CRPD3 values correlated with the time of first gas passing and the length of the hospital stay (Electronic Supplementary Material, Fig. 2b and 2c).

We utilized CRPD3 as a surrogate measure that allowed us to quantitatively estimate the degree to which the RPG method could reduce total surgical trauma, representing a novel application of CRPD3. Using linear regression analysis, we determined that RPG instead of CPG could reduce CRPD3 by approximately 6% among patients undergoing gastrectomy (Electronic Supplementary Material, Fig. 1). However, the potential benefits of RPG diminish with more extensive gastrectomy and LND. Therefore, our study suggests that patients who require STGD1+ procedures represent the best candidates for RPG, particularly women without comorbidities undergoing SGTD1+ procedures with partial omentectomy.

Acute pain is a frequent postoperative occurrence that delays the healing process, increases complications and mortality, and increases nursing costs and the duration of hospitalization [33]. Laparoscopic or robotic instruments transmit a surgeon’s external movements within the intraperitoneal cavity. The trocar acts as a fulcrum that bears the force of movement against the peritoneal wall. The somatic pain related to trocar insertion is sharper and more acute than the visceral pain associated with resection [34]. We hypothesized that the RPG method helps reduce somatic pain by piercing two to three fewer ports than CPG. The effect of RPG on CRPD3 was significant only in the STGD1+ group (Fig. 3b); however, the impact of RPG on bowel recovery and hospital stay was significant in STGD1+, STGD2, and TGD1+ (Fig. 3c, d). Furthermore, several previous studies reported that RPG showed less operative pain than CPG [35, 36], which is presumed to have led to more ambulation in those patients. As a result, the effect of shortening the recovery period was extended to the STGD2 and TGD1+ groups, in which the reduction of CRPD3 was not significant.

In the TGD2 group, RPG showed no improvement in CRPD3, bowel recovery, and hospital stay. Visceral and somatic pain associated with surgical trauma increased with more extensive gastrectomy (partial vs. total) and LND (D1+ vs. D2) procedures [37] (Figs. 2, 3). We hypothesize that the difference between CPG and RPG is clinically negligible if total surgical trauma is extensive.

This study has some limitations. First, CRPD3 is a clinically meaningful but indirect parameter for assessing surgical trauma. Second, to develop an ideal model for determining the optimal patient group for RPG, we excluded patients with complex presentations (Clavien-Dindo ≥ 3, hospital stay ≥ 14 d). Third, we only analyzed surgical trauma without considering cosmetic effects, which are crucial for younger patients.

In conclusion, we dissected and quantitatively assessed the perioperative parameters contributing to CRPD3 and found that CRPD3 varies with the extent of gastrectomy, the range of LND, sex, comorbidities, and obesity. RPG significantly reduces CRPD3 and expedites bowel recovery and hospital stay. Patients with STGD1+ benefit the most, followed by those with STGD2 and TGD1+.

### Supplementary Information

Below is the link to the electronic supplementary material.

Supplementary file1 (PDF 253 KB)
