# Supplementary material for: Identifying the best candidates for reduced port gastrectomy
Source: Gastric Cancer. 2023 Oct 24;27(1):176–86. doi: 10.1007/s10120-023-01438-6 (PMC10761455; doi:10.1007/s10120-023-01438-6)

**Electronic supplementary figure 1. Effect ratio on estimated CRPD3**

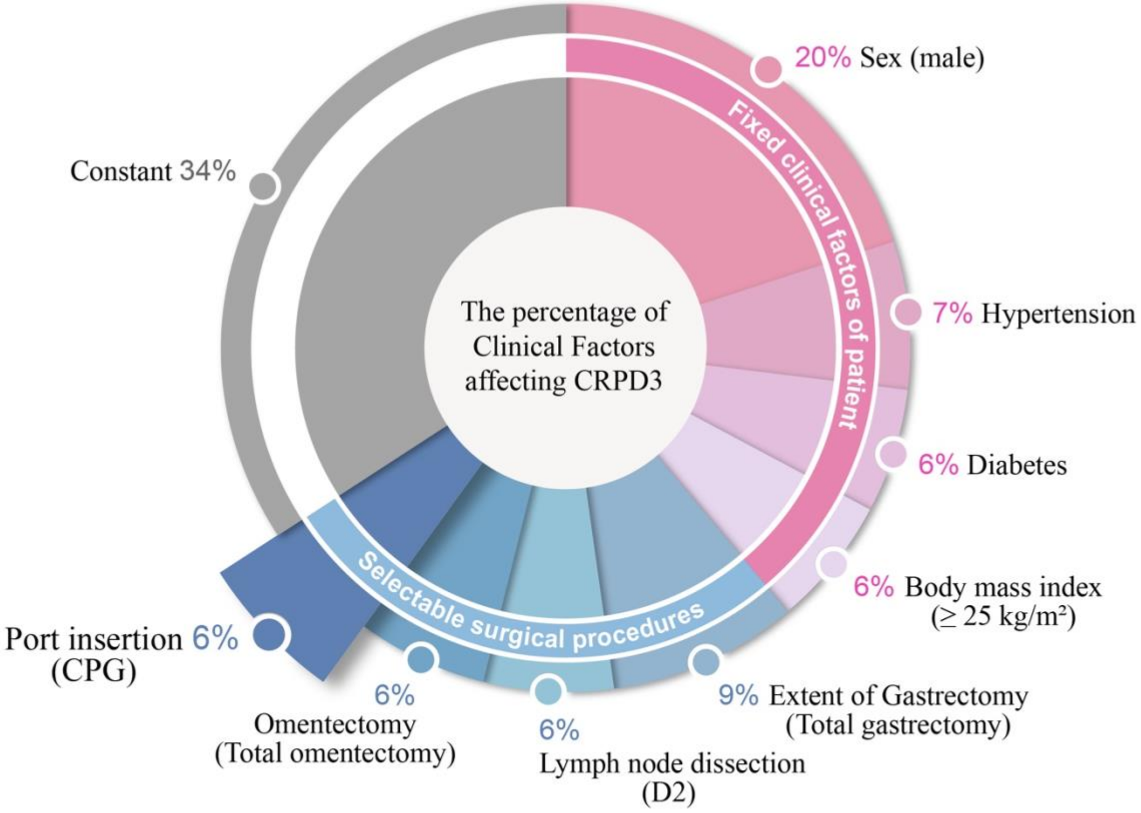


References: Sex (female), Body mass index (< 25 $kg/m^2$), Extent of gastrectomy (Subtotal gastrectomy), Lymph node dissection (D1+), Omentectomy (Partial omentectomy), Port insertion (Reduced port gastrectomy)

**Electronic supplementary figure 2. Correlation between postoperative CRP levels after gastrectomy and patient recovery**

a. **Postoperative CRP**

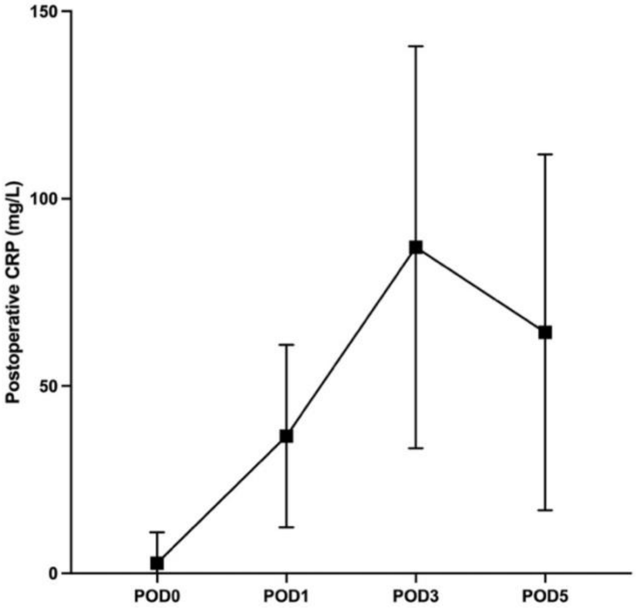


b. **The first day of gas passing and CRPD3**

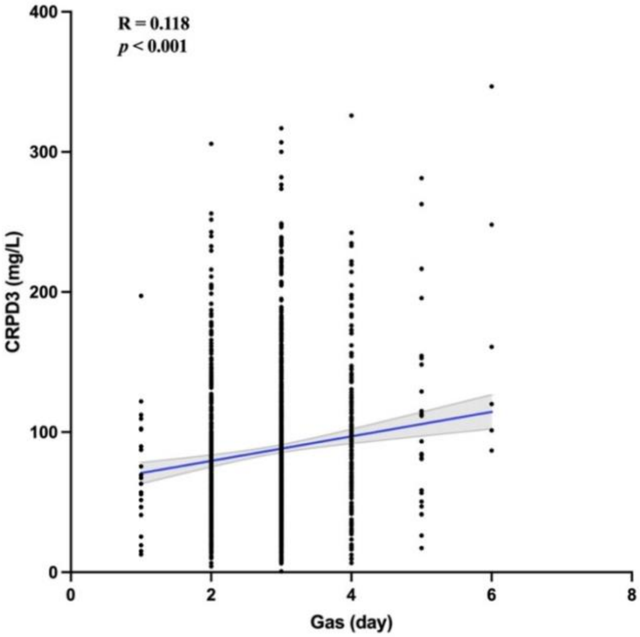

## c. Hospital stay and CRPD3

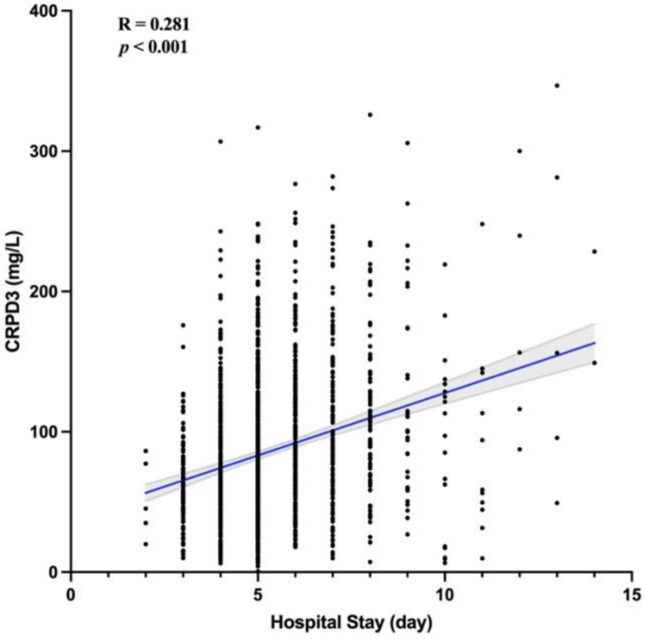

Supplement: Supplementary file 1 — Supplementary file1 (PDF 253 KB) [file 10120_2023_1438_MOESM1_ESM.pdf]
